# Optimizing Oral Vaccine Distribution Strategies for Wild Boars Through Bias-Corrected Habitat Modeling: A Case Study of Classical Swine Fever Control in Japan

**DOI:** 10.1155/tbed/1576080

**Published:** 2025-04-26

**Authors:** Satoshi Ito, Jaime Bosch, Cecilia Aguilar-Vega, Norikazu Isoda, José Manuel Sánchez-Vizcaíno, Masuo Sueyoshi

**Affiliations:** ^1^South Kyushu Livestock Veterinary Medicine Center, Kagoshima University, Soo, Japan; ^2^VISAVET Health Surveillance Center, Complutense University of Madrid, Madrid, Spain; ^3^Department of Animal Health, Faculty of Veterinary Medicine, Complutense University of Madrid, Madrid, Spain; ^4^Laboratory of Microbiology, Department of Disease Control, Faculty of Veterinary Medicine, Hokkaido University, Sapporo, Japan; ^5^Global Station for Zoonosis Control, Global Institute for Collaborative Research and Education, Hokkaido University, Sapporo, Japan; ^6^Institute for Vaccine Research and Development, Hokkaido University, Sapporo, Japan

**Keywords:** animal disease, classical swine fever, infectious disease, species distribution model, surveillance, wild boar, wildlife epidemiology

## Abstract

Control of infectious diseases in wildlife is often considered challenging due to the limited availability of information. Some infectious diseases in wildlife can also affect livestock, posing significant problems for the animal farming industry. In Japan, classical swine fever (CSF) reemerged in September 2018. Given the availability of commercial vaccines, control measures mainly involve the vaccination of domestic pigs and the distribution of oral vaccines to wild boars. Despite these efforts, the disease continues to spread, primarily due to wild boars. This transmission is further exacerbated by Japan's challenging geography—about 66% forested—making many areas difficult to access and leading to spatial bias in surveillance. As a result, the epidemic situation cannot be fully understood, limiting the effectiveness of control measures. This study estimated wild boar distribution using a species distribution model (SDM) that incorporates geographic bias correction. Two maximum entropy (MaxEnt) models—a standard model and a reporting bias-corrected model—were developed using wild boar observation data from Aichi Prefecture. Both models demonstrated excellent prediction accuracy (area under the curve [AUC] of 0.946 and 0.946, sensitivity of 0.868 and 0.943, and specificity of 0.999 and 0.991), with the most influential variables identified in a similar order (solar radiation in November, followed by elevation, precipitation during the wettest quarter, and solar radiation in August). While both models identified high-probability areas in the east, the bias-corrected model also revealed expanded high-probability zones in the northeast. During the epidemic phases, protecting farms takes priority; however, in eradication phases, control measures must also target wild boar habitats in forested areas. By using open-access environmental data, this modeling approach can be applied to other regions. Accurate estimation of wild boar distribution can contribute to improving wildlife disease surveillance and optimizing oral vaccine delivery strategies.

## 1. Introduction

Control of infectious diseases in wildlife is often considered more challenging than in livestock, as information on species distribution and population density is frequently limited. As a result, implementing intervention-based management strategies, such as vaccination, becomes challenging. A major issue for the livestock industry is that some wildlife diseases can affect livestock. When the distribution ranges of wildlife and livestock overlap, infection can spread from wildlife to domestic animals, or vice versa, through direct or indirect transmission pathways. Representative examples of these diseases include avian influenza, tuberculosis, brucellosis, anthrax, African swine fever (ASF), and Aujeszky's disease [1–3]. Therefore, establishing proper surveillance and monitoring programs for wildlife is considered crucial.

In Japan, the outbreak of classical swine fever (CSF) was confirmed in September 2018, marking the first occurrence in 26 years [4]. Caused by the CSF virus of the genus *Pestivirus* in the family Flaviviridae, this disease is classified as a notifiable disease in Japan. Infected farms are required to cull all pigs, resulting in significant economic losses. Since the disease can affect all species within the family *Suidae*, ongoing outbreaks have been reported in both domestic pigs and wild boars. Direct and indirect transmission both play crucial roles in the disease's infection cycle. Transmission occurs through direct contact between healthy and infected individuals. The virus is excreted in saliva, nasal discharge, urine, and feces; thus, contaminated vehicles, clothing, and pork derived from infected pigs are considered significant risk factors for the further spread of the infection [5].

National countermeasures for CSF in wild boars generally consist of three main components: (i) enhanced surveillance to monitor the infection status, (ii) intensified culling to reduce wild boar population density and limit further transmission, and (iii) the distribution of oral vaccines to induce immunity, thereby containing outbreaks and preventing spatial expansion [6]. Among these, oral bait vaccination for wild boars and vaccination of domestic pigs have played central roles in Japan's CSF control strategy, with field implementation beginning in March 2019 for wild boars and in October 2019 for domestic pigs [7].

This study focuses, in particular, on optimizing oral vaccine distribution, one of the key components, while also aiming to indirectly support surveillance and culling efforts through more accurate predictions of wild boar habitat distribution. Oral vaccines are distributed in collaboration with local municipalities and hunting associations. Based on expert and hunter input, bait-encased vaccines are buried at locations where wild boars are likely to appear, and additional bait is placed on top to attract the animals [8]. For example, in Aichi Prefecture, oral vaccines are distributed four times per year. Between 2018 and 2024, an average of 76,000 baits were distributed annually across ~3800 sites throughout the prefecture [8]. However, the continued spread of disease among wild boars suggests that while these measures may have reduced the viral load in the target populations, they have not yet resulted in disease eradication.

Our previous study revealed that during the vaccine distribution conducted in Gifu Prefecture from 2019 to 2020, ~70% of the oral vaccine distribution sites showed no evidence of wild boar presence or vaccine consumption [9]. Furthermore, Japan's terrain—characterized by a high forest coverage of ~66% and rugged mountainous landscapes [10]—hinders human access and results in geographic bias in surveillance activities. Consequently, it becomes difficult to accurately estimate wild boar distribution, thereby impeding proper assessment of the epidemic situation and limiting the effectiveness of control measures.

In Japan, wild boars are considered to inhabit all regions of the country except Hokkaido [11]. As omnivores, they have a varied diet, feeding on plant roots and tubers by digging up the ground, as well as acorns, insects, and reptiles [12–14]. Although they are generally considered to have high reproductive potential, reports indicate that piglet survival rates are unstable, leading to significant fluctuations in population size [15]. They typically give birth to an average of four piglets in the spring, with a single breeding cycle per year in Japan [16].

Building on these findings, we also conducted an analysis to identify optimal vaccine distribution sites based on the provided vaccine distribution location data, wild boar consumption data, and camera data. The results identified key factors for selecting vaccine distribution sites and provided spatial information for prioritizing distribution areas [9, 17]. However, considering that many municipalities have limited information beyond the geographic coordinate data of observed wild boars, this approach has limitations in its applicability to other areas. Primarily, the fundamental issue lies in the absence of wild boars at vaccine distribution sites, making it essential to accurately understand their habitat distribution.

Species distribution models (SDMs) are tools commonly used to predict species' habitat distributions by combining observations of their presence with environmental estimates. These models are widely used across terrestrial, freshwater, and marine domains, employing various approaches such as generalized linear models, random forests, and support vector machines [18]. Maximum entropy (MaxEnt) model is one of the most popular methods, as it can model species' geographic distributions using presence-only data [19].

In this study, we developed a method for estimating wild boar habitat distribution using a MaxEnt model, based on geographic coordinates of observation points and publicly available environmental data, with the aim of enabling its application to other regions. The MaxEnt model, which handles presence-only data, has significant limitations due to sample selection bias [20]. To address this, we corrected for reporting bias in wild boar observations caused by geographic factors, thereby providing accurate habitat distribution predictions and contributing to disease control.

## 2. Materials and Methods

### 2.1. Study Area

**Figure 1:**
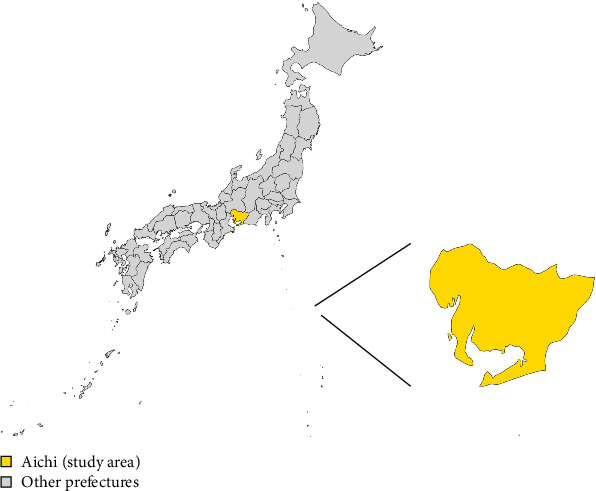
Aichi Prefecture is located in the central part of Japan (indicated in yellow on the map).

In this study, the shapefile for Aichi Prefecture was obtained from the GADM database [21] and set as the study area (Figure 1). Located in the central part of Japan, the western region of the prefecture is home to a large metropolitan area centered around Nagoya, with the Nobi Plain extending across the area. The eastern region features the Toyohashi Plain, extending to the Atsumi Peninsula, while the northern part is a mountainous area with several mountain ranges. The climate is generally mild, with the mountainous areas receiving more annual rainfall compared to the plains and peninsula regions [22]. Aichi, which hosts one of Japan's largest pig farming areas, has reported infections in both wildlife and livestock hosts since the confirmation of CSF-positive wild boars in 2018. As of the end of November 2024, 18 out of the 94 reported farm infections nationwide have occurred within the prefecture. Some areas with a high risk of contact between susceptible animals have made disease control in wild boar populations an urgent priority.

### 2.2. Collection and Preprocessing of Environmental Variables

The following environmental variables were selected for the prediction of wild boar distribution. These variables were chosen based on previous studies [9, 17, 23] and obtained from international open databases: road density, water density, human population density, elevation, slope, solar radiation, bioclimatic variables, and Normalized Difference Vegetation Index (NDVI). A brief description of the selected variables and the reference databases is summarized in Table 1. To ensure consistent processing of the wild boar observation data and environmental variables, all data were resampled to a unified resolution of ~1 km^2^ (corresponding to ~ 0.0089° latitude × 0.0089° longitude, based on the WGS84 geographic coordinate system; EPSG:4326) in the R programming environment. The multicollinearity between variables was checked using Pearson's correlation coefficient, and highly correlated variables were removed to eliminate redundancy. All variables were standardized prior to analysis to ensure comparability across different scales. The following operations were all executed in R, utilizing the following packages: *sf*, *raster*, *dplyr*, *stringr*, *viridis*, *osmdata*, *terra*, *ggplot2*, *dismo*, *reshape2*, *car*, *gridExtra*, and *sp*.

### 2.3. Collection of Wild Boar Observation Data and Correction of Spatial Bias (Spatial Thinning)

The geographic spatial data of wild boars captured or discovered during surveillance activities for CSF from November 2018 to April 2024 were provided by Aichi Prefecture. To ensure reliable distribution estimates, it was necessary to construct a more uniform and representative dataset. In this study, spatial bias caused by spatial autocorrelation and multiple observations was minimized by removing duplicate data observed within the same grid [29].

### 2.4. Background Data

The MaxEnt model is well-known for predicting species distributions using only presence data; however, in reality, background data (pseudo-absence data) is required instead of absence data. In this study, background data for the Aichi Prefecture area was extracted through a two-step process as described next. Since the home range of wild boars is significantly influenced by landscape features and the urgency of the situation, grids within a 5 km radius from the observation points were excluded to account for individual movement and contact risk [30, 31]. Furthermore, we adopted the Quality of Available Habitats for Wild Boar (QAH) map, proposed by Bosch et al. [32], as the scale for the wild boar habitat. This map, developed for the entire Eurasian continent, quantifies the suitability of land for wild boar habitats in seven categories (0, 0.1, 0.5, 1, 1.5, 1.75, and 2). The applicability of this map to Aichi Prefecture was evaluated by examining the correlation between each QAH value and the average wild boar observation density. According to Bosch et al. [32], regions with a QAH value of 1 or higher are generally considered suitable for wild boar habitation. Therefore, 10,000 random background data points were generated from areas with a QAH value of 0.5 or lower [33].

### 2.5. Running the Standard MaxEnt Model

The distribution of wild boars was predicted using the *dismo* package in R, based on the environmental variables, observation data, and background data prepared through the above process. 70% of the data was used for model training, while the remaining 30% was used for validation.

### 2.6. Construction of MaxEnt Model With Reporting Bias Correction: Estimation and Adjustment of Biased Regions

We then performed wild boar distribution predictions considering reporting bias. In a previous study conducted in South Korea, elevation and distance from roads were identified as major factors influencing surveillance activities, likely due to the impact of steep mountainous terrain [34]. Given the similar topographical characteristics of Japan [35], the present study followed a comparable approach to estimate areas likely affected by reporting bias. First, the relationships between wild boar observation counts and the two environmental variables—elevation and distance from roads—were examined using Pearson's correlation coefficient, confirming strong negative correlations for both variables. Next, to understand how these variables are distributed specifically at wild boar observation locations and to determine appropriate thresholds for identifying potential under-surveilled areas, gamma, normal, and log-normal distributions were fitted to each dataset using the *fitdistrplus* package in R. The best-fitting distribution for each variable was selected based on the Akaike Information Criterion (AIC), and the 95th percentile of each selected distribution was used as the threshold. This percentile was chosen because 90% was considered too inclusive and 97.5% too conservative, following deliberation among the authors. Regions where either distance to roads or elevation exceeded their respective 95th percentile thresholds, and where the QAH value was 1 or higher, were defined as under-surveilled areas. This condition is expressed as:

(*A* ∪ *B*) ∩ *C*,

where *A* = {Distance to roads ≥ 95th percentile}, *B* = {Elevation ≥ 95th percentile}, and *C* = {QAH ≥ 1}.

These areas are considered likely habitats for wild boars that have not been adequately covered by surveillance efforts.

### 2.7. Construction of MaxEnt Model With Reporting Bias Correction: Generation of Corrected Presence Data

We identified areas that are both suitable habitats for wild boars (with QAH values of 1 or higher) and areas where surveillance has been consistently conducted (no reporting bias areas), which are referred to hereafter as “suitable survey areas.” The average wild boar observation density in these areas was then calculated. The obtained observation density was multiplied by the area of the reporting bias regions to estimate the potential wild boar observations that were not detected during the study period in those areas. These estimates were then randomly distributed within the reporting bias regions. The resulting data, combined with the original wild boar observation data, were defined as the corrected presence data, accounting for reporting bias.

### 2.8. Running the MaxEnt Model With Reporting Bias Correction

Background data for 10,000 locations were generated under the same conditions as in the standard MaxEnt model execution, with the same environmental dataset applied to run the model.

### 2.9. Model Evaluation and Visualization of Wild Boar Predicted Distribution

The predictive accuracy of both models was evaluated based on area under the curve (AUC), sensitivity, and specificity. Using the *ggplot2* package, the predicted wild boar distributions from both models were visualized, and the contribution of each variable to the model was also plotted.

To facilitate comparison of spatial prediction patterns of the two models, we classified the predicted probability of wild boar presence into three levels (low, medium, and high) using the Jenks natural breaks method implemented in the *ClassInt* package in R. Classification thresholds were determined based on the output of the standard MaxEnt model and then uniformly applied to the bias-corrected model.

Furthermore, to quantitatively evaluate differences in predicted probabilities between the two models, we compared pixel-level probability values using the Wilcoxon signed-rank test. This nonparametric test was chosen to account for the paired nature of the data and potential non-normality in the distribution of probability values.

## 3. Results

The summary statistics of all variables used in this study are provided in Table S1. Variable pairs with a Pearson correlation coefficient of 0.7 or higher were identified as potentially having multicollinearity. Variables selected based on the authors' expertise were then used in subsequent analyses (Table 1).

Data on wild boar observations from 5898 locations during the study period were used for the analysis. The correlation between each QAH value and the average wild boar observation density in Aichi Prefecture was examined, revealing a Pearson correlation coefficient of 0.733, indicating a strong positive correlation.

### 3.1. Standard MaxEnt Model

**Figure 2:**
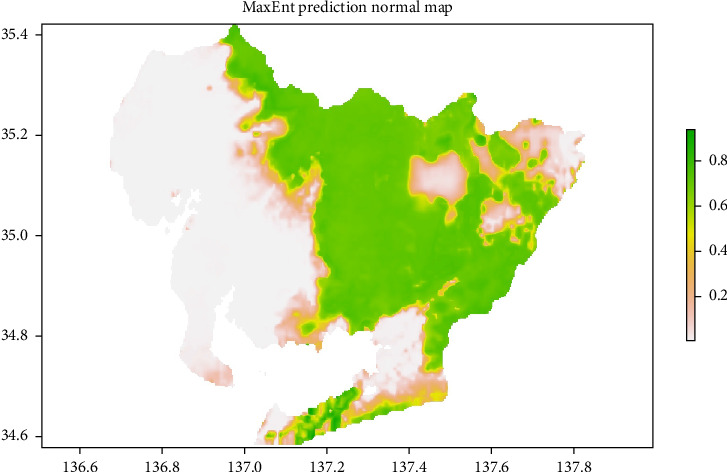
Wild boar distribution prediction map generated by the standard MaxEnt model based on data observed during the study period in Aichi Prefecture. The likelihood of wild boar habitation is color-coded between 0 and 1. Green areas represent regions with a high likelihood of wild boar habitation, while gray areas indicate regions with a low likelihood of habitation. MaxEnt, maximum entropy.

For analysis, wild boar presence data were spatially thinned to 744 points, which were subsequently used for model implementation. The predicted distribution of wild boar generated by the standard MaxEnt model is shown in Figure 2. High-probability areas for wild boar habitation are primarily spread across the eastern part of the prefecture, while low-probability areas are located in the northeastern part.

**Figure 3:**
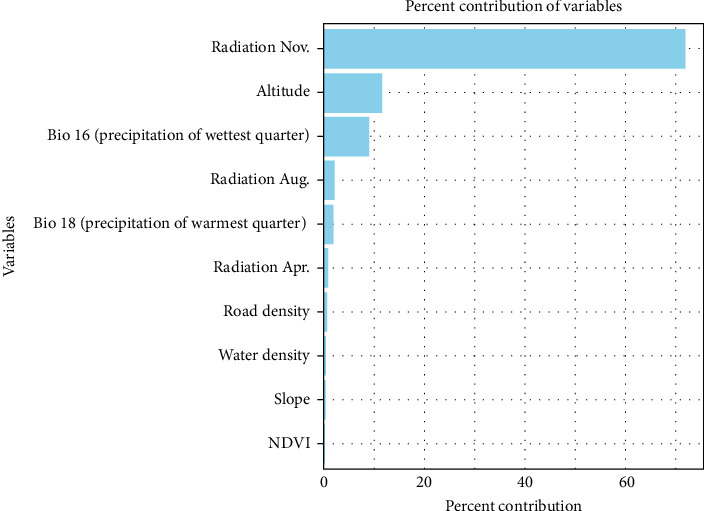
Top 10 most important variables contributing to the standard MaxEnt model. MaxEnt, maximum entropy.

The AUC, sensitivity, and specificity of this model were 0.946, 0.868, and 0.999, respectively, indicating a highly accurate predictive model. Solar radiation in November was identified as the most influential factor in the model, followed by elevation, precipitation during the wettest quarter, and solar radiation in August (Figure 3).

### 3.2. MaxEnt Model With Reporting Bias Correction

**Figure 4:**
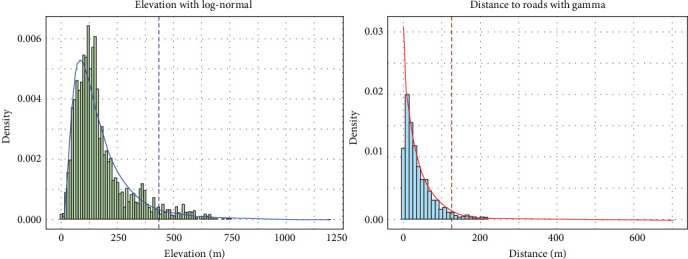
Fitted log-normal and gamma distributions to elevation and distance from roads, respectively, based on values at wild boar observation sites. The dotted lines indicate the 95th percentile thresholds.

Pearson's correlation analysis revealed a very strong negative relationship between wild boar observation counts and both elevation and distance from roads, with correlation coefficients of −0.852 and −0.880, respectively. The distributional analysis showed that the gamma distribution best fit the distance to roads data, while the log-normal distribution provided the best fit for elevation, based on AIC. Both variables exhibited right-skewed distributions. Based on the best-fit distributions of the elevation and road distance values recorded at wild boar observation sites, the 95th percentile thresholds were calculated and identified as 125.5 m for distance from roads and 432.8 m for elevation (Figure 4).

**Figure 5:**
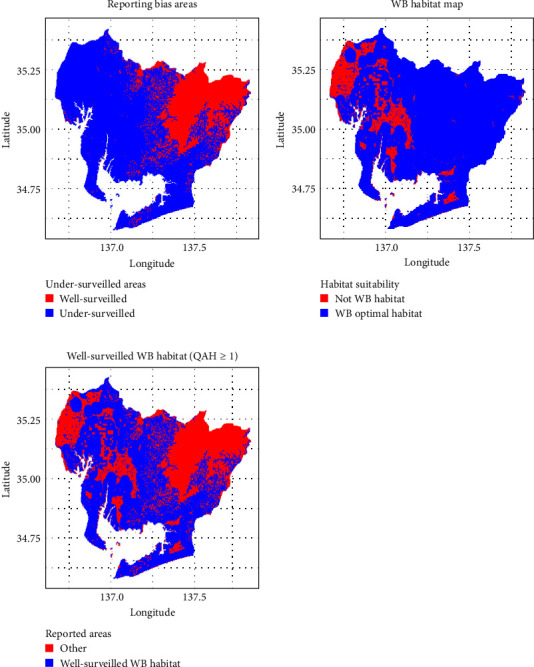
From top left, top right, to bottom left: a map showing the reporting bias areas (regions with elevation greater than 432.8 m and distance from roads greater than 125.5 m, and QAH value of 1 or higher), a map showing the optimal wild boar habitat (QAH value of 1 or higher), and a map showing the suitable survey areas (areas with a QAH value of 1 or higher where surveillance has been adequately conducted). QAH, quality of available habitats.

**Figure 6:**
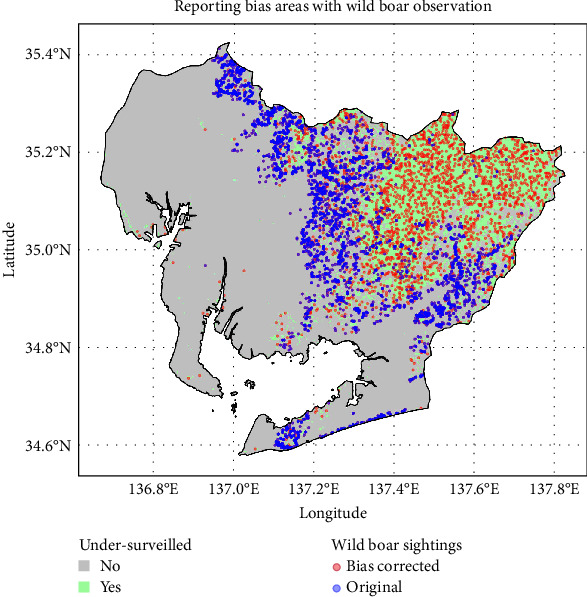
The wild boar observation points provided by Aichi Prefecture and the bias-corrected observation points are shown in blue and red, respectively. The green areas indicate regions affected by reporting bias.

Combining these results with the QAH map (areas with QAH values of 1 or higher), reporting bias areas and suitable survey areas within Aichi Prefecture were estimated (Figure 5). The average number of wild boar observations per grid in the suitable survey areas during the study period was 0.134 heads/grid, with a minimum of 0, a maximum of 31, and a standard deviation of 0.86. Based on this result, the estimated potential number of wild boar observations in the reporting bias areas was 2238. The spatial distributions of the original observations and the bias-corrected observations are shown in blue and red, respectively, on a map (Figure 6).

**Figure 7:**
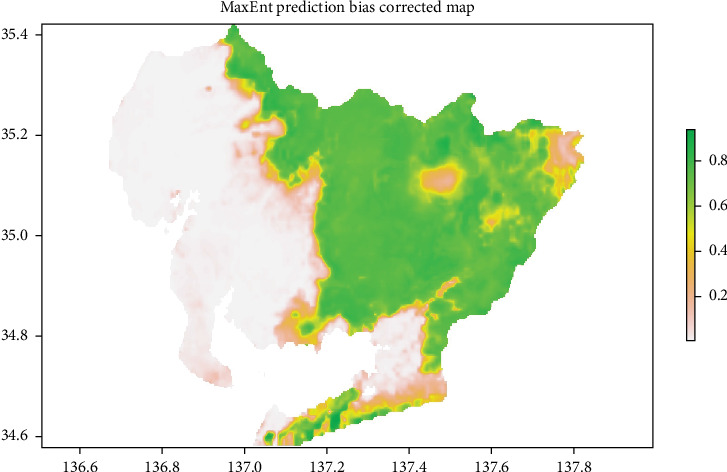
Wild boar distribution prediction map based on the MaxEnt model with reporting bias correction. The likelihood of wild boar habitation is color-coded between 0 and 1. The color scale is the same as in Figure 2. MaxEnt, maximum entropy.

The combined dataset of original wild boar observation points and those generated through bias correction totaled 8136, which was spatially thinned to 1179 points and subsequently used as presence data for model implementation. The wild boar distribution prediction obtained by the MaxEnt model with reporting bias correction is shown in Figure 7. Similar to the standard MaxEnt model, high-probability areas for wild boar habitation are mainly concentrated in the eastern part of the prefecture. Notably, in the northeastern part of the prefecture, areas with a higher likelihood of wild boar habitation have expanded compared to the standard MaxEnt model.

The proportions of each probability level before and after bias correction are presented in Table 2. Note that this is a relative classification by Jenks natural breaks method. After bias correction, the high-probability area expanded by 4.62%. The Wilcoxon signed-rank test confirmed a statistically significant shift in probability distribution before and after bias correction (*p*< 2.2e–16), indicating a meaningful impact on the overall predicted probabilities.

**Figure 8:**
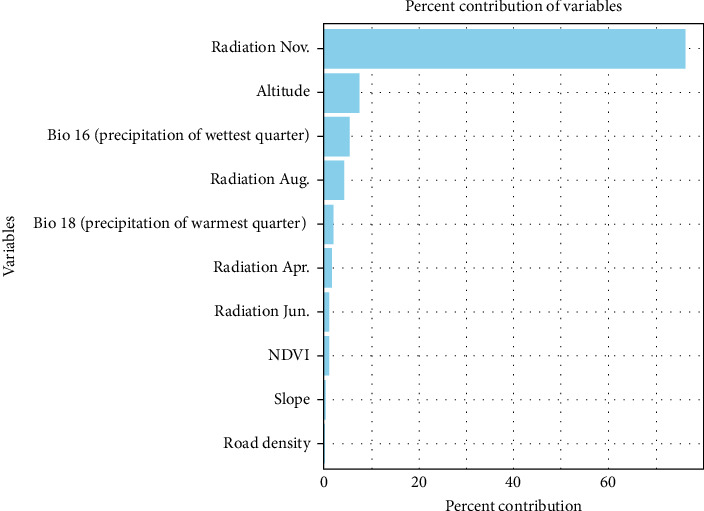
Top 10 most important variables contributing to the MaxEnt model with reporting bias correction. MaxEnt, maximum entropy.

The AUC, sensitivity, and specificity of this model were 0.946, 0.943, and 0.991, respectively, indicating a highly accurate predictive model. Similar to the standard MaxEnt model, solar radiation in November was identified as the most influential factor in this model, followed by elevation, precipitation during the wettest quarter, and solar radiation in August (Figure 8).

## 4. Discussion

The effective control of CSF in wild boars through oral vaccination can be conceptualized as a four-step process. The first step is the availability of a highly effective vaccine, the second is the targeted distribution of the vaccine based on the epidemic situation, the third is ensuring that wild boars consume the distributed vaccine, and the fourth is evaluating this entire process, identifying issues, and making improvements. This study primarily focused on the second point.

As of the end of October 2024, CSF has been confirmed in 39 out of 47 prefectures in Japan. The infected areas continue to expand, and with this, the target areas for vaccine distribution are also growing [36]. At present, the oral vaccine for wild boars is dependent on imports from Germany, resulting in a limited domestic supply. Therefore, enhancing vaccine distribution efficiency has become an urgent priority.

This study addressed the issue of spatial bias that often accompanies the implementation of SDMs[37, 38]. Two approaches were incorporated to improve the accuracy of wild boar habitat distribution estimates: spatial thinning and correction of potential observation numbers in reporting bias areas. A comparison of the two resulting models revealed significant differences, particularly in the northeastern region. This area, identified as a reporting bias region in Figure 5, comprises the municipalities of Shinshiro City, Shitara Town, Toei Town, and Toyone Village, collectively known as Oku-Mikawa, and accounts for ~20% of Aichi Prefecture's total area. Despite its large geographic size, it is sparsely populated, with less than 1% of the prefecture's population (~55,000 people as of 2015), and around 90% of its land area is covered by forests. Geographically, the region borders Nagano and Gifu Prefectures to the north and Shizuoka Prefecture to the east [39, 40]. Due to its remote, sparsely populated nature and extensive forest coverage, it is presumed to have limited surveillance coverage and shares forested areas with neighboring prefectures while having few pig farms.

These findings underscore the importance of tailoring disease control strategies to the geographical and demographic realities, as well as the epidemiological situation, of each region. For example, areas such as Oku-Mikawa may not be prioritized during the epidemic phase, when the main objective is to protect pig farms from infection. However, in the eradication phase, such under-surveilled, forest-dense regions become increasingly relevant, as they may sustain undetected transmission cycles. In Japan, where a chain of mountainous regions runs longitudinally through the country, these inaccessible areas are widespread [10], presenting a major challenge for long-term control of CSF in wild boar populations. The bias-corrected SDM developed in this study offers a practical framework for identifying such regions and can serve as a valuable tool to guide more geographically inclusive and phase-specific disease control strategies.

Among all predictors, solar radiation in November emerged as the most influential factor in both models. This variable was originally included to reflect its influence on ecological and behavioral cycles in wild boars. Solar radiation not only affects vegetation growth and temperature but also plays a significant role in regulating hormonal rhythms and reproductive timing in animals [41]. In Japan, wild boars generally enter their breeding season around November, during which males expand their movement range in search of females [42]. Additionally, the onset of winter alters the availability of food resources, causing wild boars to roam more widely in search of sustenance. This seasonal increase in movement may also be influenced by age structure within the population. Wild boars commonly give birth in the spring, and juveniles typically begin solitary movement by summer, reaching an age when they actively explore new areas by autumn [41]. These biological patterns, combined with environmental drivers such as temperature and vegetation dynamics, likely contribute to increased detectability and clearer differentiation between suitable and unsuitable habitats during the late autumn season. Another possible contributing factor is the onset of the hunting season, which typically begins around November and continues through February or March in many regions. Although the direct effect of hunting on spatial behavior is difficult to quantify, increased human activity during this period may induce behavioral adaptations, including increased movement range or altered activity patterns in some individuals, potentially contributing to broader observation ranges.

Altitude was the second most influential factor. It can serve as a physical constraint on movement and indirectly influence temperature and vegetation composition, both of which affect habitat suitability for wild boars. Precipitation during the wettest quarter was the third most important variable. Seasonal increases in water availability influence the distribution of key resources such as drinking spots and wallowing sites. Some wild boars may change their preferred resource areas frequently, which can expand their movement range and increase variability in presence data.

Solar radiation in August was also identified as an influential factor, further supporting the notion that seasonal dynamics play a critical role in shaping wild boar distribution. These findings underscore the potential value of incorporating temporal variation into habitat modeling to improve the accuracy of disease surveillance and to inform more effective control strategies. Additionally, land use types—such as forest, agricultural, and urban areas—are ecologically important determinants of wild boar habitat suitability [43, 44]. Data on capture efforts may also enhance model accuracy by providing context for observed presence patterns. If such datasets become consistently available across regions in Japan, future models could achieve greater predictive reliability and practical utility for disease management.

One limitation of this study is that the potential wild boar observations in reporting bias areas were estimated based on detection rates from standard surveillance areas. There is currently a lack of useful knowledge regarding differences in population density between mountainous regions and areas closer to human settlements. Even within areas with similar habitat suitability, the actual population density of wild boars is likely to vary, which could influence the prioritization of management measures. Although this study focused on habitat distribution rather than density estimation, we acknowledge that population density represents another important aspect of effective disease management, including strategic decisions such as the prioritization of oral vaccine distribution.

ASF, which also infects both wild boars and domestic pigs, is known as a disease that shares similarities with CSF [45]. In European countries and South Korea, wild boars are considered key drivers of infection spread [46, 47], and SDM-based approaches, such as carcass searches, have been incorporated to support surveillance efforts [48, 49]. Despite the lack of a proven, highly effective ASF vaccine, some European countries have successfully eradicated the disease from wild boar populations. Belgium demonstrated that a combination of zoning, carcass searches, depopulation, and fencing can successfully eradicate ASF in wild boar populations, even in the absence of a highly effective ASF vaccine [50]. Terrain-related surveillance bias presents a major obstacle to effective CSF and ASF control. By integrating bias correction into habitat modeling, this study provides a practical and scalable tool that can support more strategic allocation of limited resources for oral vaccination and surveillance—not only in Japan, but in other countries facing similar geographic challenges.

## Figures and Tables

**Table 1:** Environmental variables used in the MaxEnt model and their sources.

Variable	Description	Selected for MaxEnt model?	Source
Road density	The density per 1 km^2^ was calculated using the R packages *sf* and *raster* from the obtained road data.	Yes	[24]

Water density	The density per 1 km^2^ was calculated using the R packages *sf* and *raster* from the obtained water flow data (Ex. rivers and secondary rivers).	Yes	[24]

Human population density	The population density per 1 km^2^	Yes	[25]

Elevation	Possibly affect the behavior range and habitat of wild boars.	Yes	[26]

Slope	Possibly affect the habitat range of wild boars.	Yes	([26]

Monthly solar radiation	A dataset representing the amount of solar energy reaching the Earth's surface.	Partially: April, June, and November.	[27]

Bioclimatic variables	Represents annual and quarterly trends in temperature and precipitation, seasonality, and extreme factors. For more details, visit (https://www.worldclim.org/data/bioclim.html).	Partially: BIO1,7,8,12,13,16,18, and 19.	[27]

NDVI (Normalized Difference Vegetation Index)	An indicator representing the amount and vitality of plants.	Yes	[28]

**Table 2:** Comparison of risk areas before and after bias correction in the MaxEnt model based on Jenks natural breaks.

Probability level (threshold)	Before (%)	After (%)
Low (0–0.168)	45.51	42.45
Medium (0.168–0.505)	10.52	8.96
High (0.505–1)	43.97	48.59

## Data Availability

The data used in this study are not publicly available and were kindly provided by Aichi Prefecture. However, some of the surveillance data is partially available online (https://www.pref.aichi.jp/soshiki/nogyo-shinko/kensa.html).

## Supporting Information

Supporting Information Table S1. Prestandardized summary statistics for each variable used in this study. Each variable is presented with its description, unit, minimum and maximum values, mean, and standard deviation.
